# Graphitic Carbon-Coated FeSe_2_ Hollow Nanosphere-Decorated Reduced Graphene Oxide Hybrid Nanofibers as an Efficient Anode Material for Sodium Ion Batteries

**DOI:** 10.1038/srep23699

**Published:** 2016-04-01

**Authors:** Jung Sang Cho, Jung-Kul Lee, Yun Chan Kang

**Affiliations:** 1 Department of Materials Science and Engineering, Korea University, Anam-Dong, Seongbuk-Gu, Seoul 136-713, Republic of Korea; 2 Department of Chemical Engineering, Konkuk University, 1 Hwayang-dong, Gwangjin-gu, Seoul 143-701, Republic of Korea

## Abstract

A novel one-dimensional nanohybrid comprised of conductive graphitic carbon (GC)-coated hollow FeSe_2_ nanospheres decorating reduced graphene oxide (rGO) nanofiber (hollow nanosphere FeSe_2_@GC–rGO) was designed as an efficient anode material for sodium ion batteries and synthesized by introducing the nanoscale Kirkendall effect into the electrospinning method. The electrospun nanofibers transformed into hollow nanosphere FeSe_2_@GC–rGO hybrid nanofibers through a Fe@GC–rGO intermediate. The discharge capacities of the bare FeSe_2_ nanofibers, nanorod FeSe_2_–rGO–amorphous carbon (AC) hybrid nanofibers, and hollow nanosphere FeSe_2_@GC–rGO hyrbid nanofibers at a current density of 1 A g^−1^ for the 150th cycle were 63, 302, and 412 mA h g^−1^, respectively, and their corresponding capacity retentions measured from the 2nd cycle were 11, 73, and 82%, respectively. The hollow nanosphere FeSe_2_@GC–rGO hybrid nanofibers delivered a high discharge capacity of 352 mA h g^−1^ even at an extremely high current density of 10 A g^−1^. The enhanced electrochemical properties of the hollow nanosphere FeSe_2_@GC–rGO composite nanofibers arose from the synergetic effects of the FeSe_2_ hollow morphology and highly conductive rGO matrix.

The demand for large-scale energy storage has increased with the development of electric vehicles (EVs) and renewable energy storage^1^,^2^,^3^,^4^. Up to now, lithium ion batteries (LIBs) have been regarded as promising candidates for these applications^5^,^6^,^7^. However, according to the rapidly growing demands for low-cost energy storage, sodium ion batteries (SIBs) may be a feasible alternative because of abundant Na resource (Na is the fourth most abundant element on earth, ~2.6% by weight of the earth’s crust) and low cost (cost equivalents in dollars for bulk metal: Na, 0.075; Li, 0.50)^8^,^9^,^10^. However, the larger Na^+^ ionic radius (0.76 Å for Li^+^ vs 1.02 Å for Na^+^) and molar mass (22.99 g/mol for Li vs 6.94 g/mol for Na) are critical obstacles that affect Na^+^ diffusion, resulting in lower specific capacities, lower rate capabilities, larger volume changes, and shorter cycling lives^11^,^12^,^13^.

To overcome these issues, developing efficient anode materials for application in SIBs with good electrochemical performance is urgently needed and desirable. Thus far, one-dimensional (1D) nanomaterials have been recognized as the most desirable materials for applications in energy storage^14^,^15^,^16^,^17^. Their unique structures provide enhanced surface-to-volume ratios, short transport lengths for Li^+^ ionic transport, and efficient 1D electron transport along their longitudinal directions^14^,^15^,^16^,^17^. Hollow structures also have exhibited good electrochemical properties at high current densities because of their decreased ion diffusion lengths and the increased contact areas between the electrolyte and electrode for Li^+^ insertion–desertion^18^,^19^,^20^,^21^,^22^. Moreover, the void spaces in such structures could accommodate the volume change during cycling^23^,^24^,^25^. However, larger Na^+^ ionic radius compared to that of Li^+^ seems to prevent the achievement of sufficiently good electrochemical properties of anode materials in SIBs even when the advantageous effects of the 1D nanomaterials and the hollow structure are combined. To surmount this, construction of a stable artificial coating layer on the surface of the hollow structure would be another effective strategy for further maintaining the structural integrity via accommodating the huge volume variations induced by Na^+^ diffusion^26^. Herein, the conductive graphitic carbon (GC) could be applied as a suitable coating layer that plays the role mentioned above and it could derivatively serve as fast and continuous transport pathways for electrons upon cycling due to its high electroconductivity. Additionally, the stable GC coating layer on the hollow structure triggers stable solid electrolyte interphase (SEI) formation which is usually formed in cycling owing to the reductive decomposition of organic electrolytes. Along with these structural strategies, graphene is considered as the most promising matrix to support anode materials in SIBs due to its prominent advantages, such as its superior electrical conductivity, high specific surface area, structural flexibility, and chemical stability, all of which are responsible for improved electrochemical performance in SIBs^27^,^28^,^29^,^30^. In this context, hybrid nanomaterials consisting of highly conductive graphene and the hollow structure with GC coating layer is expected to be a tactic for efficient anode materials in SIBs with good electrochemical performance.

Herein, we designed 1D nanomaterials comprising graphitic carbon (GC)-coated hollow metal chalcogenide nanospheres decorated within a reduced graphene oxide (rGO) nanofiber (hollow nanosphere FeSe_2_@GC–rGO). Novel-structured rGO nanofibers comprising graphitic carbon-coated FeSe_2_ hollow nanospheres selected as the first target material were prepared by applying the nanoscale Kirkendall diffusion process to the conventional electrospinning process in order to create this anode material for SIBs, although there has been scarce study concerning the electrochemical-reaction mechanism of FeSe_2_ for SIBs. The electrochemical properties of the FeSe_2_@GC–rGO hybrid nanofibers comprising FeSe_2_ hollow nanospheres were compared with those of the bare FeSe_2_ nanofibers and FeSe_2_–rGO–amorphous carbon (AC) hybrid nanofibers comprising FeSe_2_ nanorods.

## Results and Discussion

**Figure 1:**
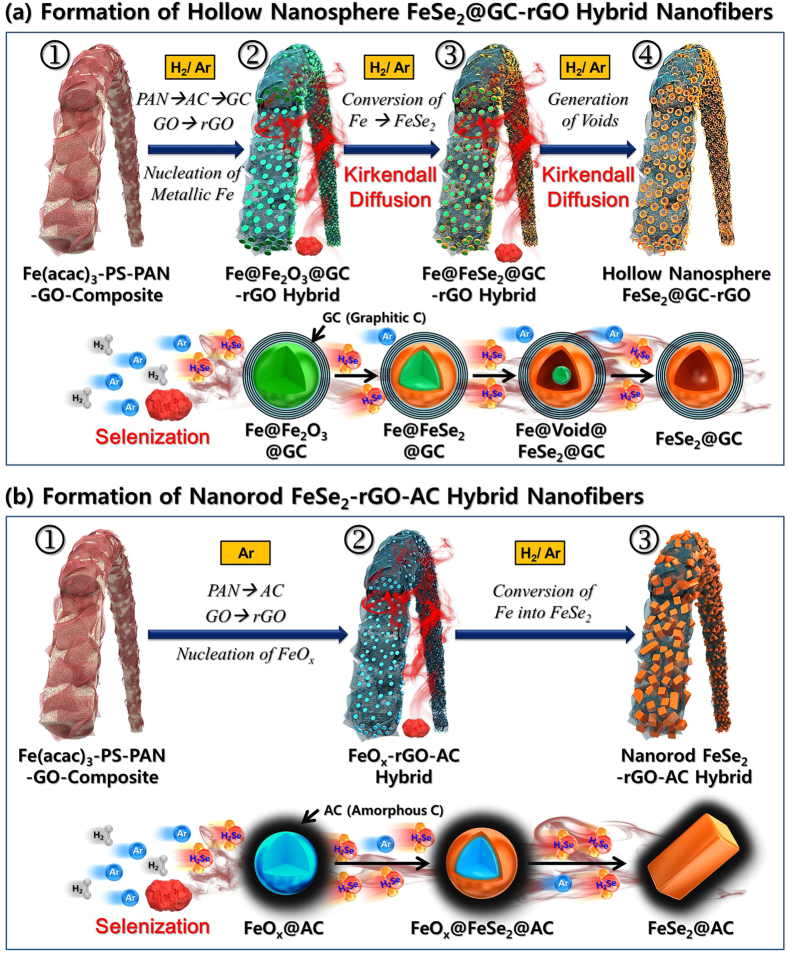
Schematic diagram for the formation mechanism of (**a**) hollow nanosphere FeSe_2_@GC–rGO and (**b**) nanorod FeSe_2_-decorated rGO-AC hybrid nanofibers.

**Figure 2:**
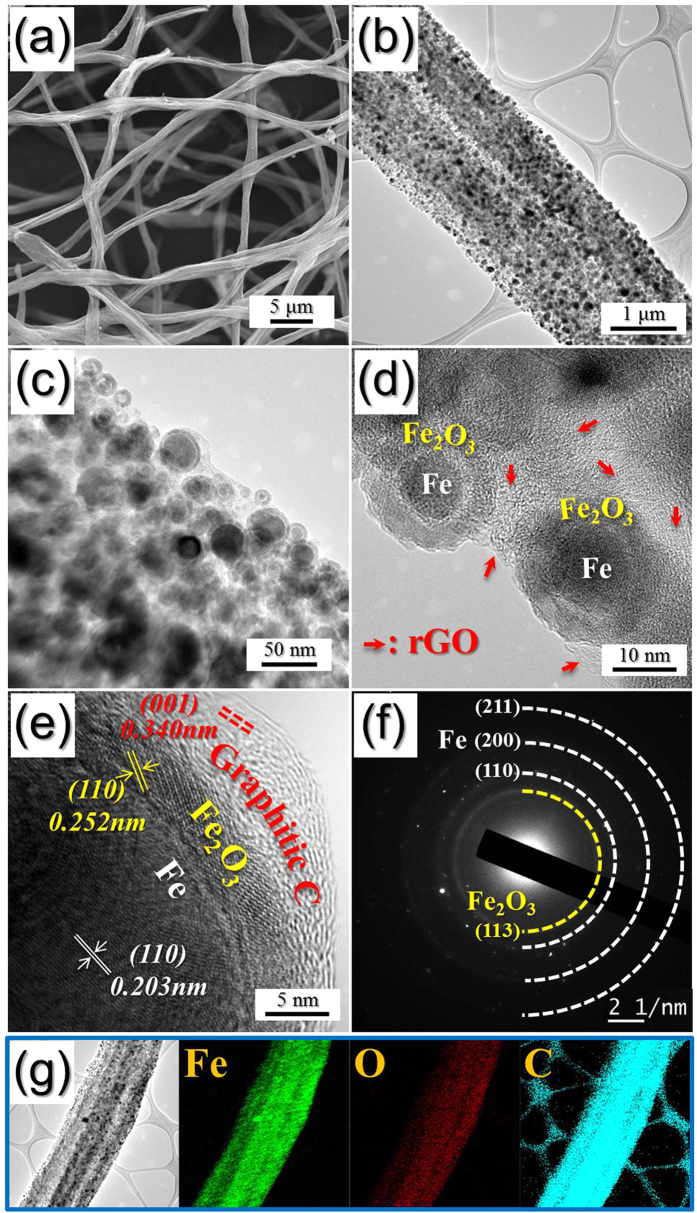
Morphologies, SAED pattern, and elemental mapping images of the Fe@GC–rGO hybrid nanofibers: (**a**) SEM image, (**b–d**) TEM images, (**e**) HR-TEM image, (**f**) SAED pattern, and (**g**) elemental mapping images.

**Figure 3:**
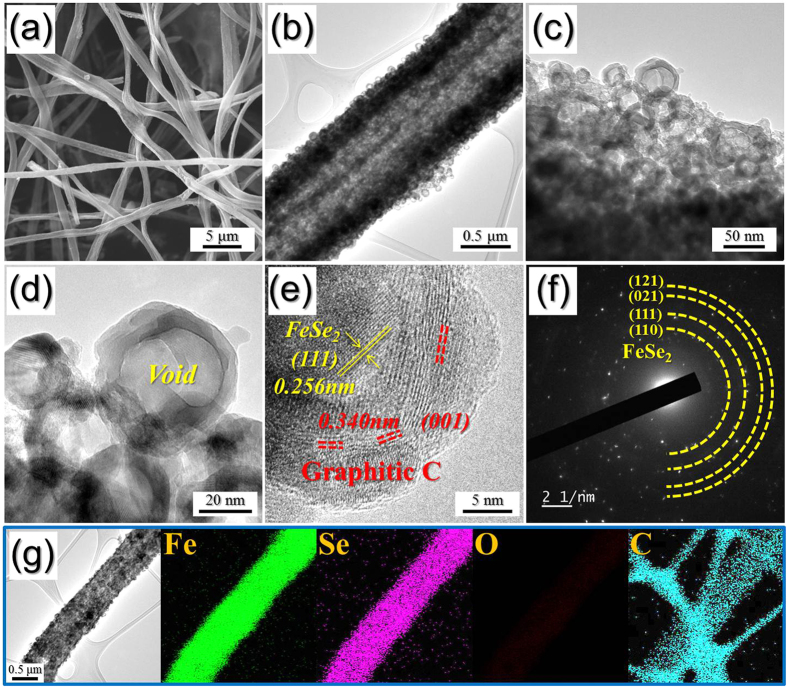
Morphologies, SAED pattern, and elemental mapping images of the hollow nanosphere FeSe_2_@GC–rGO hybrid nanofibers: (**a**) SEM image, (**b–d**) TEM images, (**e**) HR-TEM image, (**f**) SAED pattern, and (**g**) elemental mapping images.

**Figure 4:**
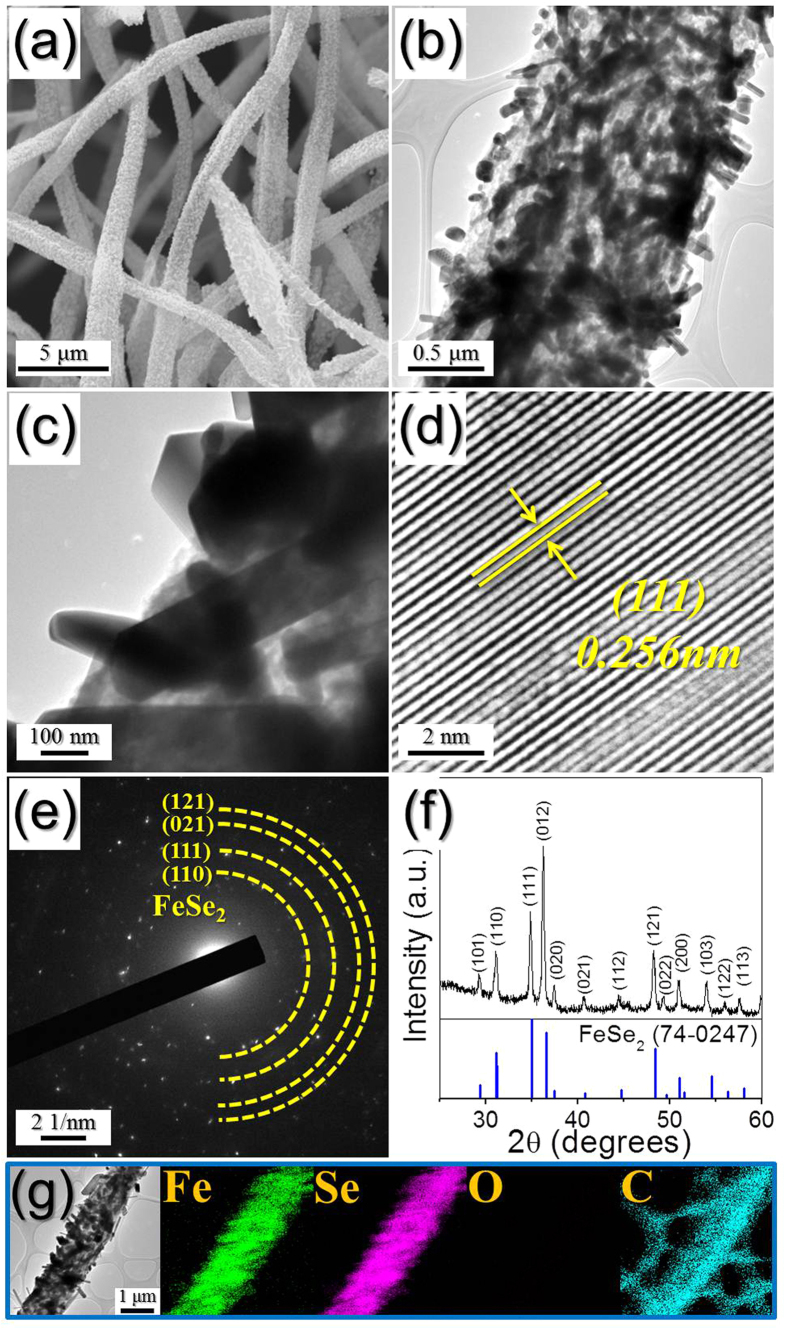
Morphologies, SAED pattern, XRD pattern, and elemental mapping images of the nanorod FeSe_2_-rGO-AC hybrid nanofibers: (**a**) SEM, (**b,c**) TEM images, (**d**) HR-TEM image, (**e**) SAED pattern, (**f**) XRD pattern, and (**g**) elemental mapping images.

The formation mechanism of the hollow nanosphere FeSe_2_@GC–rGO hybrid nanofibers is described in Fig. 1a. The composite nanofibers composed of PAN, PS, GO, and Fe(acac)_3_ were prepared via an electrospinning process (Fig. 1a-①). A reduction process of these electrospun nanofibers under a H_2_/Ar atmosphere at 500 °C produced the metallic Fe@GC–rGO hybrid nanofibers (Fig. 1a-②). Reduction of the GO nanosheets into rGO and carbonization of PAN into amorphous carbon (AC) material occurred during this reduction process. The AC covering the Fe nanocrystals changed into graphitic carbon (GC) during reduction, a process for which the metallic Fe nanocrystals acted as a catalyst. Decomposition of PS into gases even under the H_2_/Ar atmosphere resulted in metallic Fe–rGO–GC hybrid nanofibers with a minimum content of amorphous carbon with low electrical conductivity and a high initial irreversible-capacity loss. The metallic Fe–rGO–GC hybrid nanofibers transformed into hollow nanosphere FeSe_2_@GC–rGO hybrid nanofibers via a selenization process under H_2_Se gas, which was formed from Se powders via a reaction with H_2_ gas (Fig. 1a-③). The Fe nanocrystals with solid structures comprising the hybrid nanofibers transformed into the FeSe_2_ hollow nanospheres via the well-known nanoscale Kirkendall diffusion process [Fe cations diffused outward more quickly than Se anion diffused inward, which is consistent with the larger ionic radius of Se anions (Se^2−^ is 184 pm) than Fe cations (Fe^2+^ is 76 pm, Fe^3+^ is 65 pm, Fe^4+^ is 72 pm)] through intermediate yolk–shell-structured nanopowders with the configuration Fe@void@FeSe_2_ (Fig. 1a-③,④). The formation mechanism of the nanorod FeSe_2_–rGO–AC hybrid nanofiber is shown in Fig. 1b. The carbonization process of the composite nanofibers composed of PAN, PS, GO, and Fe(acac)_3_ under Ar atmosphere at 500 °C produced the FeO_x_–rGO–AC hybrid nanofibers (Fig. 1b-①,②). The selenization of FeO_x_ nanocrystals formed the FeSe_2_ nanorods surrounded by amorphous carbon (AC) (Fig. 1b-③). In this case, graphitization of the amorphous carbon did not occur due to the absence of a metallic Fe catalyst.

The morphologies of the electrospun nanofibers are shown in Fig. S1a. The composite nanofibers composed of PAN, PS, GO, and Fe(acac)_3_ with a mean diameter of 3 μm had filled structures and smooth surfaces. The morphologies of the metallic Fe@GC–rGO hybrid nanofibers obtained after the reduction process are shown in Fig. 2. The electrospun nanofibers transformed into nanofibers with porous and withered structures via the reduction process. The TEM images shown in Fig. 2b,c reveal the ultrafine nanopowders uniformly dispersed within the hybrid nanofibers. The nanopowders shown in Fig. 2d had yolk–shell structures with the configuration Fe@void@Fe_2_O_3_. The exposure of ultrafine Fe nanocrystals under an air atmosphere at room temperature after the reduction process formed the Fe_2_O_3_ layer via surface oxidation. The diffusion out of the Fe component into the surface of the nanopowders via nanoscale Kirkendall diffusion formed the nanopowders with the yolk–shell structures. The nanopowder shown in the high-resolution TEM image in Fig. 2e had a double–shelled structure with the configuration Fe@Fe_2_O_3_@GC (graphitic carbon). The graphitic carbon formed due to the Fe catalyst uniformly covering the Fe@Fe_2_O_3_ nanopowder. The high-resolution TEM image shown in Fig. 2e revealed clear lattice fringes separated by 0.203, 0.252, 0.340 nm, which correspond to the (110), (110), and (001) crystal planes of Fe, Fe_2_O_3_, and graphitic carbon phase, respectively. The SAED and XRD patterns shown in Figs 2f and S2, respectively, revealed the formation of the Fe@GC–rGO hybrid nanofibers. The SAED pattern revealed the presence of (110), (200), and (211) lattice planes corresponding to the Fe phase, as well as (113) plane of Fe_2_O_3_ phase due to the partial surface oxidation of Fe nanocrystals by exposure in air. The elemental-mapping images shown in Fig. 2g revealed the uniform distribution of the Fe nanocrystals over the hybrid nanofibers. The trace amounts of oxygen due to surface oxidation of the Fe nanocrystals was observed from the elemental-mapping image.

The morphologies of the hollow nanosphere FeSe_2_@GC–rGO hybrid nanofibers formed via the selenization process of the metallic Fe@GC–rGO hybrid nanofibers are shown in Fig. 3. The overall morphology of these nanofibers did not change during the selenization process. However, the inner structure of the nanofibers observable in the TEM images strictly changed with the selenization process. The hybrid nanofibers were composed with hollow nanospheres formed via the nanoscale Kirkendall diffusion process. The high-resolution TEM image shown in Fig. 3e revealed clear lattice fringes separated by 0.256 and 0.340 nm, which corresponded to the (111) and (001) crystal planes of FeSe_2_ and graphitic carbon, respectively. The SAED and XRD patterns shown in Figs 3f and S2, respectively, revealed the formation of the FeSe_2_@GC–rGO hybrid nanofibers. The Fe-to-Se component ratio observed from the energy dispersive spectroscopy (EDS) analysis shown in Fig. S3 was approximately 2. The Raman spectrum shown in Fig. S4 contained the characteristic wide *D* and *G* bands of carbon around 1340 and 1590 cm^−1^, respectively. The higher signal peak intensity of the D band compared to that of the G band is indicative of the thermal reduction of GO nanosheets to rGO nanosheets during the post-heat-treatment process^31^. The elemental-mapping images shown in Fig. 3g revealed the uniform distribution of the FeSe_2_ hollow nanospheres over the hybrid nanofibers.

The chemical state and molecular environment of the metallic Fe@GC–rGO and hollow nanosphere FeSe_2_@GC–rGO hybrid nanofibers were characterized via X-ray photoelectron spectroscopy (XPS). In the Fe 2*p* spectrum of the metallic Fe@GC–rGO hybrid nanofibers shown in Fig. S5a, there were two peaks at binding energies of 711 eV for Fe 2*p*_3/2_ and 724 eV for Fe 2*p*_1/2_, which are the characteristic peaks of Fe(III) in *a*-Fe_2_O_3_ due to the partial surface oxidation of the Fe nanocrystals by exposure in air^14^. In the Fe 2*p* spectrum of the hollow nanosphere FeSe_2_@GC–rGO hybrid nanofibers shown in Fig. S5b, the main peaks observed occurred at binding energies of 706 eV for Fe 2*p*_3/2_ and 720 eV for Fe 2*p*_1/2_; these are characteristic of FeSe_2_ and a shake-up satellite^32^. In the Se 3*d* spectrum in Fig. S5c, the binding energies at 54.55 eV for Se 3*d*_5/2_ and 55.31 eV for Se 3*d*_3/2_ were also confirmed to be in good agreement with the reported values for FeSe_2_ in the literature^32^. Additionally, the Se–O bond observed at 58.2 eV revealed the existence of a small amount of SeO_2_ impurities formed during the selenization process. The C 1*s* peak shown in Fig. S5d revealed peaks corresponding to *sp*^2^-bonded carbon (C–C), epoxy and alkoxy groups (C–O), and carbonyl and carboxylic (C=O) components at 284.6, 286.6, and 288.1 eV, respectively^10^. The C–C bond peak was strong, while the C–O and C=O peaks were weak, indicating the thermal reduction of the GO nanosheets to rGO nanosheets during the two-step post-treatment preparation process. The TG curve of the hollow nanosphere FeSe_2_@GC–rGO hybrid nanofibers shown in Fig. S6 revealed a one-step weight increase and two-step weight loss for temperatures below 600 °C. The partial oxidation reaction of FeSe_2_ with oxygen resulted in a weight increase around 230 °C. The two-step weight loss observed at temperatures between 250 and 500 °C was attributed to the decomposition of FeSe_2_ into Fe_2_O_3_ and the combustion of rGO and GC. The rGO–GC content of 27% was estimated from the TG analysis of the hollow nanosphere FeSe_2_@GC–rGO hybrid nanofibers.

Bare FeSe_2_ and nanorod FeSe_2_–rGO–AC hybrid nanofibers as the comparison samples were also prepared via a selenization process of the bare Fe_2_O_3_ and FeO_x_–rGO–AC hybrid nanofibers, respectively. The bare Fe_2_O_3_ nanofibers shown in Fig. S7 were formed via the oxidation of the electrospun nanofibers under an air atmosphere at 500 °C. The bare Fe_2_O_3_ nanofibers with cubic structure transformed into bare FeSe_2_ nanofibers with orthorhombic structure via the selenization process shown in Figs S7 and S8. The ultrafine Fe_2_O_3_ nanocrystals transformed into the submicron-sized FeSe_2_ crystals during the selenization process. The high-resolution TEM image shown in Fig. S8d revealed clear lattice fringes separated by 0.287 nm, which corresponded to the (110) crystal plane of FeSe_2_ phase. The SAED and XRD patterns shown in Fig. S8e,f, respectively, revealed the formation of the phase pure FeSe_2_ nanofibers. In addition, the elemental-mapping images shown in Fig. S8g revealed the formation of carbon-free FeSe_2_ nanofibers.

The nanorod FeSe_2_–rGO–AC hybrid nanofibers prepared via the selenization of the FeO_x_–rGO–AC hybrid nanofibers also had a unique structure. The FeSe_2_ nanorods were uniformly embedded within the rGO–AC nanofibers, as shown by the SEM and TEM images in Fig. 4a–c. The mean thickness of the FeSe_2_ nanorods measured from the TEM images was 148 nm. The high-resolution TEM image shown in Fig. 4d revealed clear lattice fringes separated by 0.256 nm, which corresponded to the (111) crystal plane of FeSe_2_ phase. The SAED and XRD patterns shown in Fig. 4e,f, respectively, revealed the formation of the FeSe_2_–rGO–AC hybrid nanofibers with a pure crystal structure of the FeSe_2_ phase. The elemental-mapping images shown in Fig. 4g revealed a uniform distribution of FeSe_2_ nanocrystals within the rGO–AC hybrid matrix. The N_2_-adsorption and -desorption isotherms and BJH pore size distributions of the three samples are shown in Fig. S9. The BET surface areas of the bare FeSe_2_, nanorod FeSe_2_-rGO-AC, and hollow nanosphere FeSe_2_@GC–rGO hybrid nanofibers were 4, 9, and 34 m^2^ g^−1^, respectively. The nanorod FeSe_2_–rGO–AC and hollow nanosphere FeSe_2_@GC–rGO hybrid nanofibers showed well-developed mesopores due to their carbon materials.

**Figure 5:**
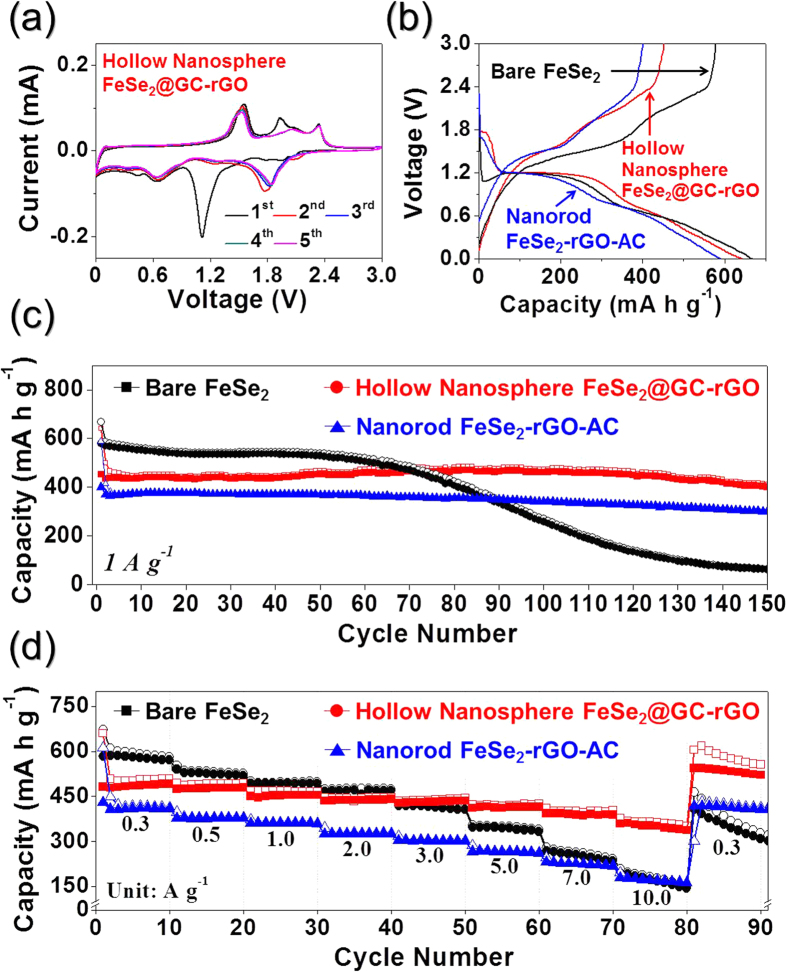
Electrochemical properties of the hollow FeSe_2_@GC–rGO hybrid, nanorod FeSe_2_–rGO–AC hybrid, and bare FeSe_2_ nanofibers: (**a**) CV curves of the hollow FeSe_2_@GC-rGO hybrid nanofibers, (**b**) first charge-discharge curves at a current density of 1.0 A g^−1^, (**c**) cycling performances at a current density of 1.0 A g^−1^, and (**d**) rate performances.

The electrochemical properties of the hollow nanosphere FeSe_2_@GC–rGO hybrid nanofibers for sodium ion storage were compared with those of the bare FeSe_2_ and nanorod FeSe_2_–rGO–AC hybrid nanofibers via cyclic voltammograms (CVs) and galvanostatic discharge–charge cycling in a voltage range of 0.001–3.0 V vs Na/Na^+^. The CVs of the hollow nanosphere FeSe_2_@GC–rGO hybrid nanofibers during the first five cycles at a scan rate of 0.07 mV s^−1^ are shown in Fig. 5a. The first cathodic scan of the hollow nanosphere FeSe_2_@GC–rGO hybrid nanofibers showed three distinct peaks located at 1.1, 0.7, and 0.4 V. The sharp reduction peak located at 1.1 V was attributed to the formation of Na_*x*_FeSe_2_^33^ and the formation of a solid electrolyte interphase (SEI) by electrolyte decomposition^34^. The two reduction peaks located at 0.7 and 0.4 V were attributed to the formations of FeSe and Na_2_Se, first, and Fe and Na_2_Se, second, respectively^33^. During the anodic scans, three oxidation peaks were observed at 1.6, 1.9 and 2.3 V, which were attributed to the formation of Na_x_FeSe_2_ and FeSe_2_, and the subsequent decomposition of the SEI layer, respectively^33^,^34^,^35^,^36^. The cathodic scans from the second cycle onward showed distinct reduction peaks at around 1.8 V. The formation of ultrafine FeSe_2_ nanocrystals during the first discharge and charge processes resulted in the reduction peak shift to a high potential. The CV curves of the bare FeSe_2_ and nanorod FeSe_2_–rGO–AC hybrid nanofibers shown in Fig. S10 had similar shapes to those of the hollow nanosphere FeSe_2_@GC–rGO hybrid nanofibers. The initial discharge and charge curves of the three samples at a current density of 1 A g^−1^ are shown in Fig. 5b. The initial discharge capacities of the bare FeSe_2_, nanorod FeSe_2_-rGO-AC, and hollow nanosphere FeSe_2_@GC–rGO hybrid nanofibers were 667, 589, and 642 mA h g^−1^, respectively, and their corresponding initial charge capacities were 578, 401, and 452 mA h g^−1^, respectively. The nanorod FeSe_2_–rGO–AC and hollow nanosphere FeSe_2_@GC–rGO hybrid nanofibers had lower initial Coulombic efficiencies than that of the bare FeSe_2_ nanofibers due to the high initial irreversible-capacity loss of the carbon material. The cycling performances of the three samples at a current density of 1 A g^−1^ are shown in Fig. 5c. The nanorod FeSe_2_–rGO–AC and hollow nanosphere FeSe_2_@GC–rGO hybrid nanofibers had superior cycling performances compared to that of the bare FeSe_2_ nanofibers. The discharge capacities of the bare FeSe_2_, nanorod FeSe_2_–rGO–AC, and hollow nanosphere FeSe_2_@GC–rGO hybrid nanofibers for the 150^th^ cycle were 63, 302, and 412 mA h g^−1^, and their corresponding capacity retentions measured from the 2^nd^ cycle were 11, 73, and 82%, respectively. The initial discharge and charge curves of the nanorod FeSe_2_-rGO-AC and hollow nanosphere FeSe_2_@GC–rGO hybrid nanofibers at a low current density of 0.05 A g^−1^ are shown in Fig. S11, in which the two samples had similar initial discharge and charge capacities. Therefore, in Fig. 5c, the fast sodium ion insertion and desertion in the hollow nanosphere FeSe_2_@GC–rGO hybrid nanofibers with their unique structure resulted in higher capacities than those of the nanorod FeSe_2_–rGO–AC hybrid nanofibers at a high current density of 1 A g^−1^. The rate performances of the three samples are shown in Fig. 5d in which the current density was increased stepwise from 0.3 to 10 A g^−1^. The hollow nanosphere FeSe_2_@GC–rGO hybrid nanofibers showed superior rate performance compared to those of the bare FeSe_2_ and nanorod FeSe_2_–rGO–AC hybrid nanofibers. The gap between the discharge capacities of the nanorod FeSe_2_–rGO–AC and hollow nanosphere FeSe_2_@GC–rGO hybrid nanofibers showing good cycling performances for sodium ion storage increased with increasing current densities. The hollow nanosphere FeSe_2_@GC–rGO hybrid nanofibers had final discharge capacities of 510, 494, 466, 448, 443, 425, 404 and 352 mA h g^−1^ at current densities of 0.3, 0.5, 1.0, 2.0, 3.0, 5.0, 7.0, and 10 A g^−1^, respectively. The discharge capacities of the hollow nanosphere FeSe_2_@GC–rGO hybrid nanofibers were well recovered to 605 mA hg^−1^ when the current density was returned to 0.3 A g^−1^ after cycling at high current densities. The formation of a polymeric gel-like film on the active material resulted in the high capacities of the hollow nanosphere FeSe_2_@GC–rGO hybrid when the current density was returned to 0.3 A g^−1^ after cycling at high current densities.

**Figure 6:**
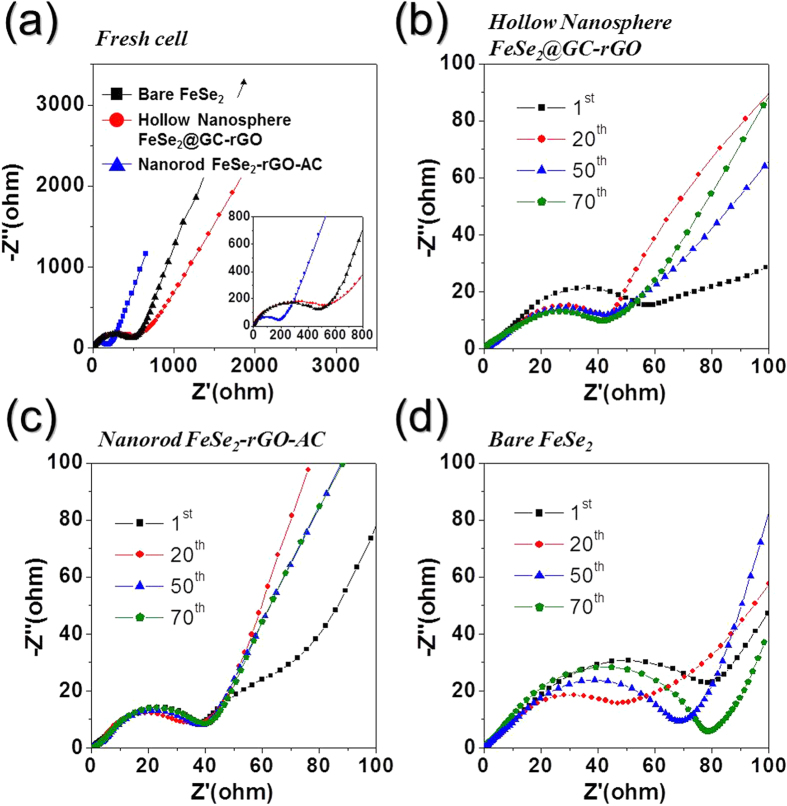
Nyquist plots of the hollow nanosphere FeSe_2_@GC–rGO hybrid, nanorod FeSe_2_–rGO–AC hybrid, and bare FeSe_2_ nanofibers: (**a**) before cycling, (**b**) after cycling of the hollow nanosphere FeSe_2_@GC–rGO hybrid nanofibers, (**c**) after cycling of the nanorod FeSe_2_–rGO–AC hybrid nanofibers, and (**d**) after cycling of the bare FeSe_2_ nanofibers.

Electrochemical-impedance-spectroscopy (EIS) measurements of the three samples were taken before and after 1, 20, 50, and 70 cycles to investigate the superior sodium ion storage properties of the hollow nanosphere FeSe_2_@GC–rGO hybrid nanofibers compared to those of bare FeSe_2_ and nanorod FeSe_2_–rGO–AC hybrid nanofibers. The Nyquist plots shown in Fig. 6 were deconvoluted with a Randle-type equivalent circuit model (Fig. S12). The equivalent circuit model describes the electrochemical reaction steps, including Na ion migration through SEI layers, a charge transfer reaction, and Na ion diffusion kinetics throughout the active materials. The nanorod FeSe_2_–rGO–AC hybrid nanofibers with high conductivity originated by AC, and lower surface area compared to hollow nanosphere FeSe_2_@GC–rGO had lower *R*_*ct*_ than those of the other two nanofibers before cycling, as shown in Fig. 6a. The *R*_*ct*_ values of the three samples decreased abruptly after the first cycle due to the transformation of the FeSe_2_ crystals into ultrafine nanocrystals during the first cycle^37^,^38^,^39^. The *R*_*ct*_ values of the bare FeSe_2_ nanofibers increased strictly during cycling, as shown in Fig. 6b. However, the *R*_*ct*_ values of the hollow nanosphere FeSe_2_@GC–rGO and nanorod FeSe_2_–rGO–AC hybrid nanofibers remained constant during 70 cycles, as shown in Fig. 6c,d, respectively. The *R*_*ct*_ values of the bare FeSe_2_, nanorod FeSe_2_–rGO–AC, and hollow nanosphere FeSe_2_@GC–rGO hybrid nanofibers after 70 cycles were 79, 41, and 41 Ω, respectively. The results of the EIS measurements revealed the structural stability of the hollow nanosphere FeSe_2_@GC–rGO and nanorod FeSe_2_–rGO–AC hybrid nanofibers during the repeated Na insertion and extraction processes. In contrast, the structural destruction during cycling increased the *R*_*ct*_ values of the bare FeSe_2_ nanofibers. Therefore, the structural stability and fast sodium insertion and desertion characteristics of the hollow nanosphere FeSe_2_@GC–rGO hybrid nanofibers correlated to their excellent Na ion storage properties.

**Figure 7:**
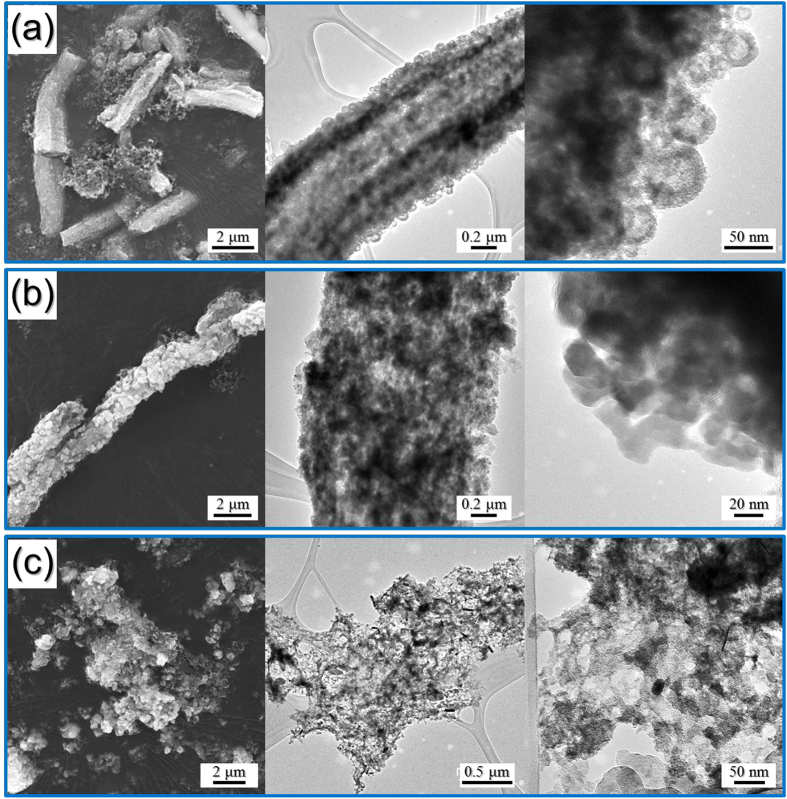
Morphologies of the (**a**) hollow nanosphere FeSe_2_@GC–rGO hybrid nanofibers, (**b**) nanorod FeSe_2_-rGO-AC hybrid nanofibers, and (**c**) bare FeSe_2_ nanofibers obtained after the 100th cycle.

The morphologies of the hollow nanosphere FeSe_2_@GC–rGO hybrid, nanorod FeSe_2_-rGO-AC hybrid, and bare FeSe_2_ nanofibers obtained after the 100^th^ cycle, are shown in Fig. 7. The hollow nanosphere FeSe_2_@GC–rGO hybrid nanofibers maintained their original morphologies well even after long-term cycling as shown by TEM images in Fig. 7a, in which the hollow structure of the FeSe_2_ nanospheres was well observed. The nanorod FeSe_2_-rGO-AC hybrid nanofibers had also maintained overall morphologies after cycling, as shown in Fig. 7b. However, the structure of the bare FeSe_2_ nanofibers completely destroyed after cycling, as shown in Fig. 7c.

**Figure 8:**
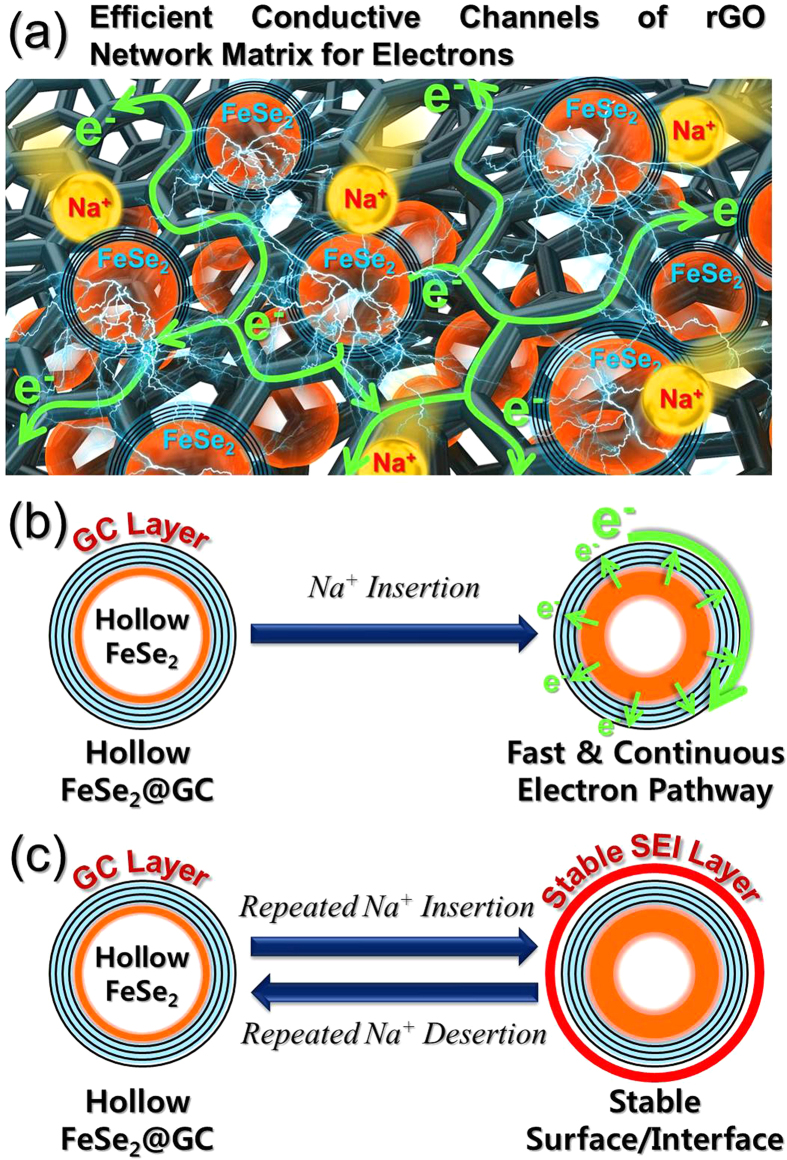
(**a**) 3-D interconnected rGO nano-networks of hollow nanosphere FeSe_2_@GC–rGO hybrid nanofibers with efficient transport pathways for electrons and Na^+^ diffusion, (**b**) illustration of electron transport pathways of GC coating layer covering the hollow FeSe_2_@GC nanosphere upon Na^+^ insertion, and (**c**) the formation of the stable SEI layer on the hollow FeSe_2_@GC nanosphere during cycling.

These enhanced electrochemical properties of the hollow nanosphere FeSe_2_@GC–rGO hybrid nanofibers arose from the synergetic effects of the highly conductive rGO nano-network matrix composing the fiber and the FeSe_2_ hollow sphere encapsulated within GC coating layer. First of all, rGO nano-network matrix surrounding the FeSe_2_@GC spheres acted as efficient conductive channels for electrons to transfer easily, as shown in Fig. 8a. Along with this, rGO nano-network matrix could act as a buffer to accommodate the volume variation of FeSe_2_@GC spheres during repeated cycling by enwrapping the spheres. In addition to the role of rGO nano-network matrix, FeSe_2_ hollow sphere with GC coating layer played at least three significant roles for further improvement in the electrochemical properties. First, FeSe_2_ hollow sphere lowered Na^+^ diffusion-induced stresses, consequently alleviating the volume variations upon Na^+^ insertion and desertion, and also had short diffusion length for Na^+^, increased contact area between the electrolyte and electrode in comparison with solid structures. Second, the highly conductive GC coating layer on the FeSe_2_ hollow sphere could primarily serve as fast and continuous transport pathways for electrons before electrons are transferred to secondary pathway of rGO nano-network matrix, as shown in Fig. 8b. It hence enabled permanent maintenance of good electrical contact with FeSe_2_ hollow spheres. Third, FeSe_2_ hollow spheres were encapsulated inside GC coating layer, which could effectively stabilize the surface of FeSe_2_ hollow sphere, thus leading to the construction of a stable solid electrolyte interphase (SEI) on it and keeping the structural integrity by additionally accommodating the huge volume expansion by Na^+^ diffusion, as shown in Fig. 8c. Simultaneously, GC coating layer inhibited the aggregation of FeSe_2_ nanospheres during repeated charge and discharge processes and thus maintaining the structural and electrical integrity of the structure. As a result, unique structure of the hollow nanosphere FeSe_2_@GC–rGO hybrid nanofibers combined the aforementioned effects of improved structural stability and maintenance of efficient electron transport pathways during long-term cycling, showing superior electrochemical performances in SIBs.

## Conclusions

We proposed the synthesis of a newly designed nanostructured material comprising graphitic carbon-coated hollow metal chalcogenide nanospheres decorated within rGO nanofibers. The hollow nanosphere FeSe_2_@GC–rGO hybrid nanofibers selected as the first target material were prepared by applying the nanoscale Kirkendall diffusion process to a conventional electrospinning process. The hollow nanosphere FeSe_2_@GC–rGO hybrid nanofibers showed superior sodium ion storage properties compared to those of the bare FeSe_2_ and nanorod FeSe_2_–rGO–AC hybrid nanofibers. The synergetic effects of the highly conductive GC-coated FeSe_2_ hollow nanospheres with high structural stability and fast and continuous transport pathways for electrons during cycling and a highly conductive rGO nano-network matrix resulted in the superior cycling and rate performances of the hollow nanosphere FeSe_2_@GC–rGO hybrid nanofibers. This simple synthesis method could be widely applied in the preparation of highly conductive GC-coated hollow metal chalcogenide nanospheres decorated within rGO nanofibers for a wide range of applications, including energy storage.

## Materials and Methods

### Sample preparation

Hollow FeSe_2_@GC–rGO hybrid nanofibers were prepared via a three-step process. For this preparation, Fe(acac)_3_-polystylene (PS)–polyacrylonitrile (PAN) with graphene oxide (GO) [Fe(acac)_3_–PAN–PS–GO] composite nanofibers were prepared as precursor fibers via an electrospinning process. GO was synthesized from graphite flakes using a modified Hummers method, as described in our previous report^40^,^41^ and then it was freeze-dried. The precursor solution for the electrospinning process was prepared by dissolving 5.0 g of Fe(acac)_3_ (STREM Chemicals, 99%), 1.0 g of PAN (Aldrich, M_w_: 150,000), 2.0 g of PS (Aldrich, M_w_: ~192,000), and 0.2 g of GO in a solution of 30 mL of N,N-dimethylformamide (DMF, Aldrich, 99%) with vigorous stirring overnight. The prepared solution was loaded at a flow rate of 2 mL h^−1^ into a plastic syringe equipped with a 25-gauge stainless steel nozzle. The solution was subsequently ejected and electrospun onto a drum collector covered with aluminum foil. During the electrospinning process, the distance between the tip and the collector was maintained at 20 cm, and the rotation of the drum was maintained at 100 rpm. The applied voltage between the collector and the syringe tip was 25 kV. The resultant Fe(acac)_3_–PAN–PS–GO composite nanofibers were stabilized at 120 °C in air for 1 h. For the FeSe_2_@GC–rGO hybrid nanofibers, the reduction process was conducted at 500 °C for 3 h in the presence of a gas mixture of 10% H_2_/Ar; this method produced metallic Fe@GC–rGO hybrid nanofibers. The subsequent selenization process was carried out at 300 °C for 6 h in H_2_Se gas, formed from commercial selenium-metal powders by hydrogen gas, to produce the hollow FeSe_2_@GC–rGO hybrid nanofibers. For the selenization process, the metallic Fe@GC–rGO hybrid nanofibers and selenium-metal powders were loaded in a covered alumina boat and placed in a quartz-tube reactor. For the FeSe_2_–rGO–AC (amorphous carbon) hybrid nanofibers comprising FeSe_2_ nanorods used for comparison purposes, as-spun Fe(acac)_3_–PAN–PS–GO composite nanofibers were post-treated at 500 °C for 3 h under an Ar atmosphere and subsequently at 300 °C for 6 h in H_2_Se gas for the selenization of the FeO_*x*_ into FeSe_2_. Bare FeSe_2_ nanofibers without carbon material were also prepared as another comparison sample. Fe(acac)_3_–PAN–PS composite nanofibers were prepared via electrospinning with identical conditions, as described above, but without containing GO in the precursor solution. The resultant Fe(acac)_3_–PAN–PS composite nanofibers were post-treated at 500 °C for 3 h under an air atmosphere and subsequently at 300 °C for 6 h in H_2_Se gas for their selenization. For simplicity, the FeSe_2_–rG–GC hybrid nanofibers comprising FeSe_2_ hollow nanospheres, FeSe_2_–rGO–AC (amorphous carbon) hybrid nanofibers comprising FeSe_2_ nanorods, and bare FeSe_2_ nanofibers are referred to as “hollow nanosphere FeSe_2_@GC–rGO”, “nanorod FeSe_2_–rGO–AC”, and “bare FeSe_2_”, respectively.

### Characterization techniques

The microstructures of the nanofibers were observed using field-emission scanning electron microscopy (FESEM, S-4800, Hitachi) and field-emission transmission electron microscopy (TEM, JEM-2100F, JEOL). In addition, their crystal structures were evaluated through X-ray diffraction (XRD, X’Pert PRO MPD) using Cu K_α_ radiation (λ = 1.5418 Å) at the Korea Basic Science Institute (Daegu). X-ray photoelectron spectroscopy (XPS, Thermo Scientific K-Alpha), with a focused monochromatic Al Kα at 12 kV and 20 mA, was used to analyze the compositions of the specimens. The surface areas of the nanofibers were determined using the Brunauer–Emmett–Teller (BET) method, where N_2_ was the adsorbate gas. The structural characterization of the carbon in the specimens was performed via Raman spectra (excited by a 632.8 nm He–Ne laser, Jobin Yvon LabRam HR800) at room temperature. Finally, thermogravimetric analysis (TGA) was performed using a Pyris 1 TGA (Perkin Elmer, temperature range = 25–650 °C, heating rate = 10 °C min^−1^, static air atmosphere).

### Electrochemical measurements

The electrochemical properties of the fabricated FeSe_2_ nanofibers were analyzed by constructing 2032-type coin cells. Each anode was prepared by mixing the active material, carbon black, and sodium carboxymethyl cellulose (CMC) in a weight ratio of 7:2:1. Na metal and microporous polypropylene film were used as the counter electrode and the separator, respectively. The electrolyte was 1 M NaClO_4_ (Aldrich) dissolved in a mixture of ethylene carbonate and dimethyl carbonate (EC/DMC, 1:1 v/v), to which 5 wt% fluoroethylene carbonate (FEC) was added. The discharge–charge characteristics of the samples were investigated by cycling the cells in a 0.001–3 V potential range at various current densities. Cyclic voltammograms (CV) were measured at a scan rate of 0.07 mV s^−1^. The dimensions of the anode were 1 cm × 1 cm, and the mass loading was approximately 1.2 mg cm^−2^. The electrochemical impedance was measured using electrochemical-impedance spectroscopy (EIS) over a frequency range of 0.01 Hz to 100 kHz by using the cell taken after fully charged up to 3.0 V.

## Additional Information

**How to cite this article**: Cho, J. S. *et al.* Graphitic Carbon-Coated FeSe_2_ Hollow Nanosphere-Decorated Reduced Graphene Oxide Hybrid Nanofibers as an Efficient Anode Material for Sodium Ion Batteries. *Sci. Rep.* **6**, 23699; doi: 10.1038/srep23699 (2016).

## Supplementary Material

Supplementary Information
