# Supplementary material for: Graphitic Carbon-Coated FeSe2 Hollow Nanosphere-Decorated Reduced Graphene Oxide Hybrid Nanofibers as an Efficient Anode Material for Sodium Ion Batteries
Source: Sci Rep. 2016 Apr 1;6:23699. doi: 10.1038/srep23699 (PMC4817055; doi:10.1038/srep23699)
Supplement: Supplementary Information [file srep23699-s1.pdf]

Supporting Information

Graphitic Carbon-Coated $FeSe_2$ Hollow Nanosphere-Decorated Reduced Graphene Oxide Hybrid Nanofibers as an Efficient Anode Material for Sodium Ion Batteries

Jung Sang Cho[1], Jung-Kul Lee[2,], and Yun Chan Kang[1,*]*

J.S. Cho, Prof. J.K. Lee and Prof. Y.C. Kang

[1]Department of Materials Science and Engineering, Korea University, Anam-Dong, Seongbuk-Gu, Seoul 136-713, Republic of Korea, E-mail: yckang@korea.ac.kr

[2]Department of Chemical Engineering, Konkuk University, 1 Hwayang-dong, Gwangjin-gu, Seoul 143-701, Republic of Korea, E-mail: jkrhee@konkuk.ac.kr

Keywords: iron selenide, sodium ion batteries, Kirkendall effect, graphene, carbon composite, electrospinning

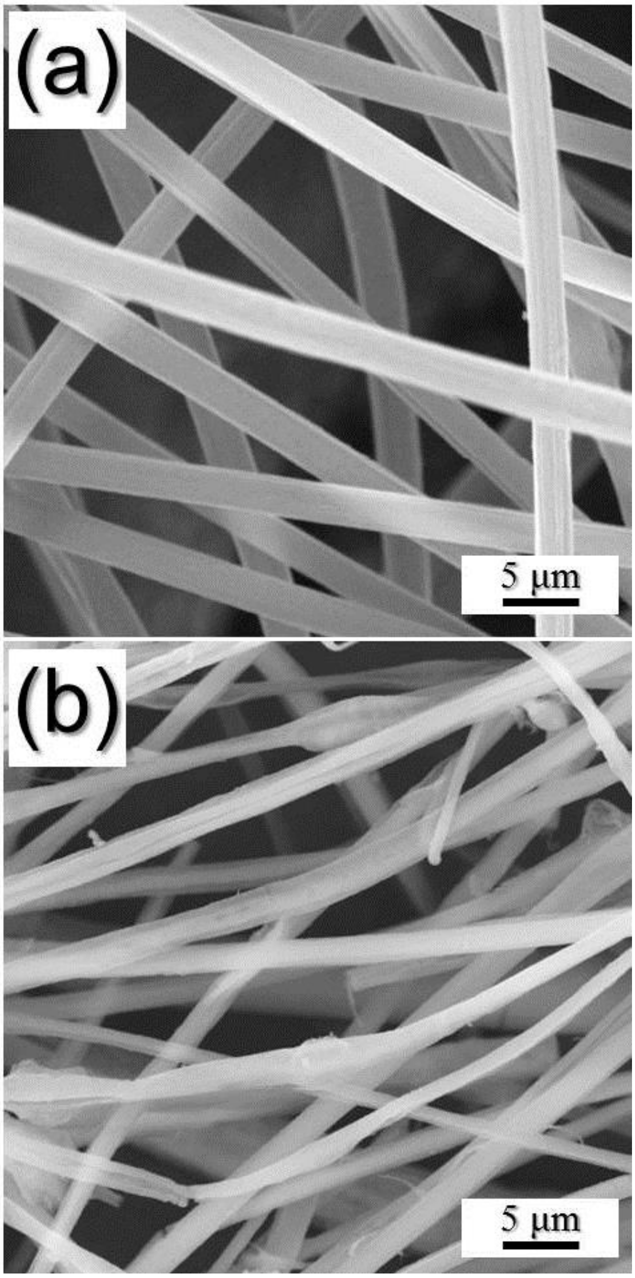

Figure S1 SEM images of the (a) as-spun $Fe(acac)_3$–PAN–PS composite nanofibers and (b) stabilized nanofibers treated at 120 ^{o}C under air atmosphere.

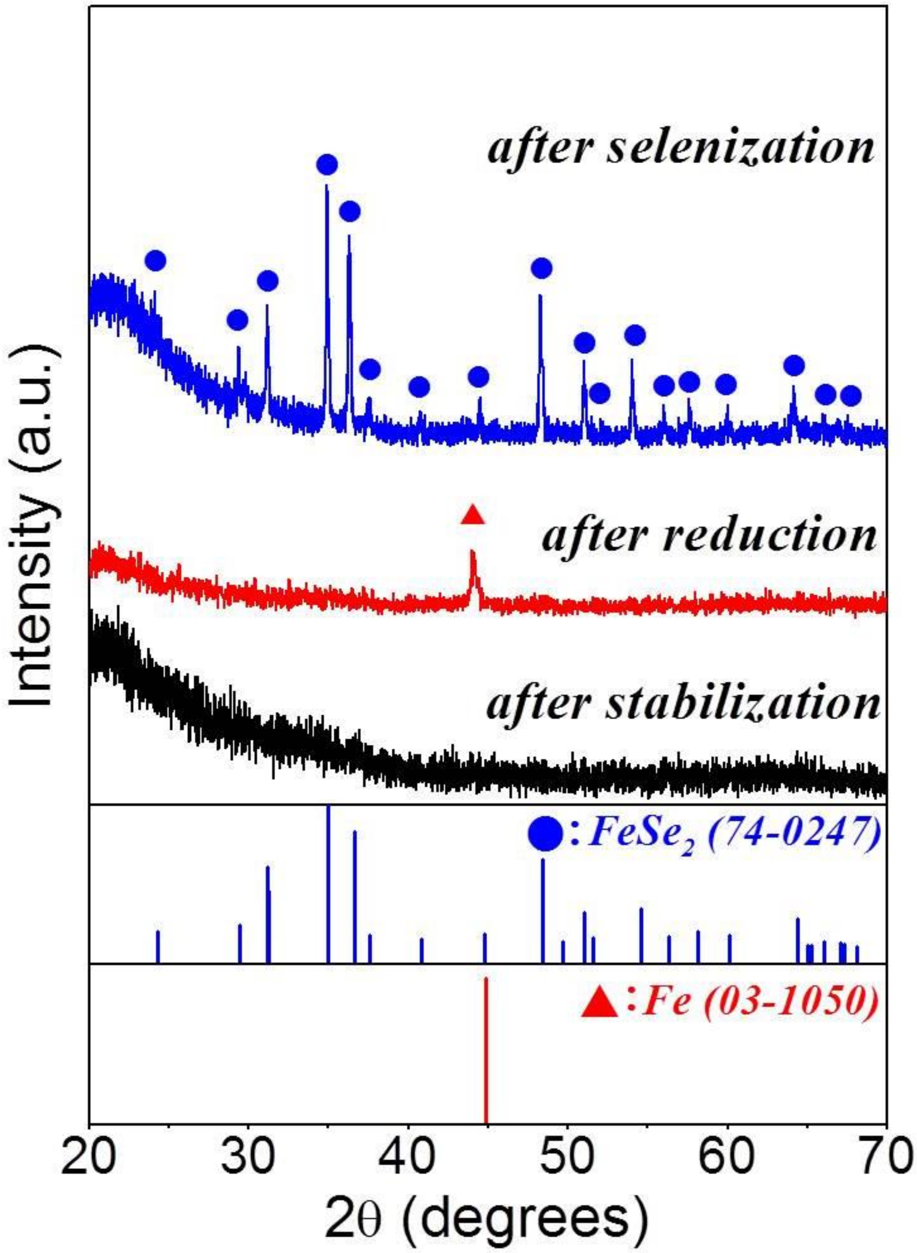

Figure S2. XRD patterns of the nanofibers obtained after stabilization process, subsequent reduction process, and final selenization process.

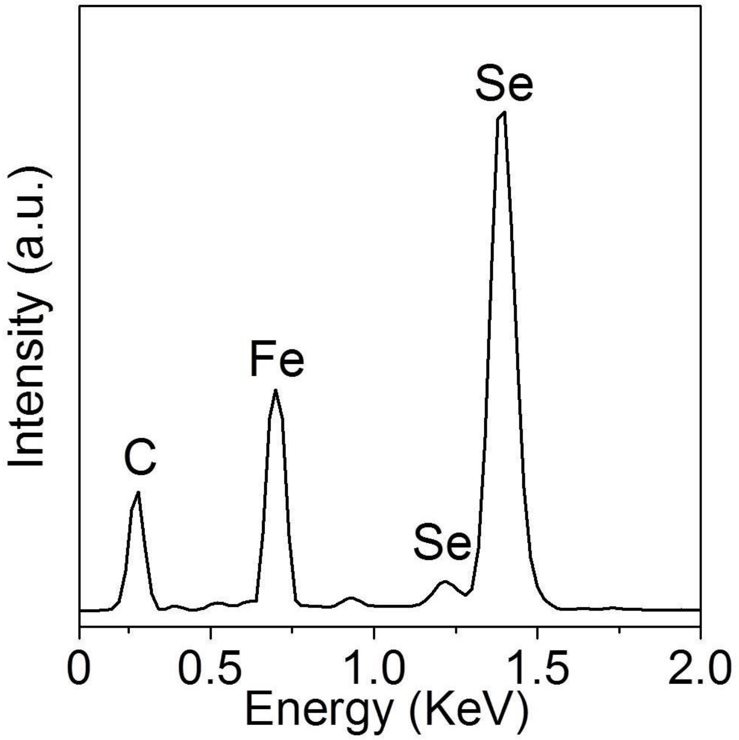

Figure S3. Energy dispersive spectroscopy (EDS) of the hollow nanosphere $FeSe_2$@GC–rGO hybrid nanofibers formed by selenization process.

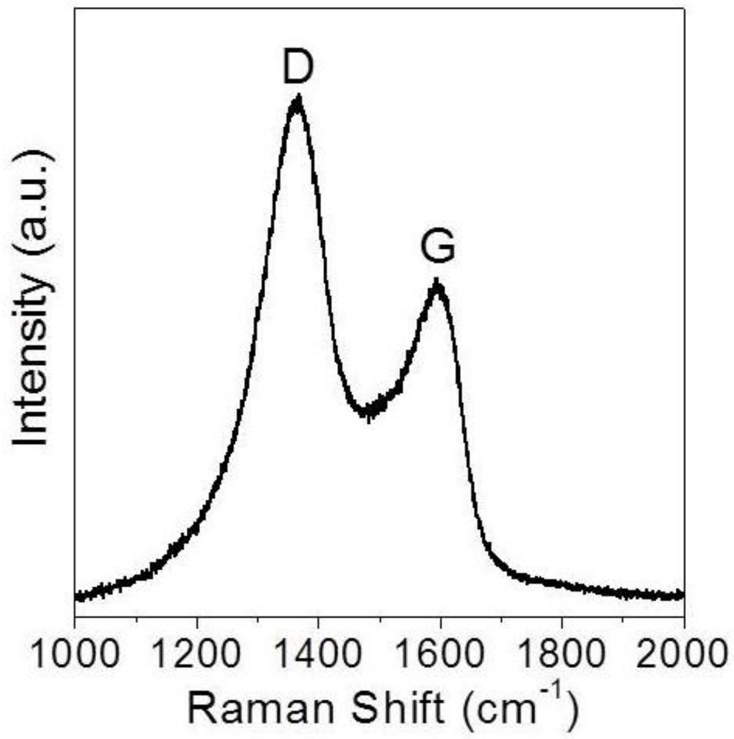

Figure S4. Raman spectrum of the hollow nanosphere $FeSe_2$@GC–rGO hybrid nanofibers formed by selenization process.

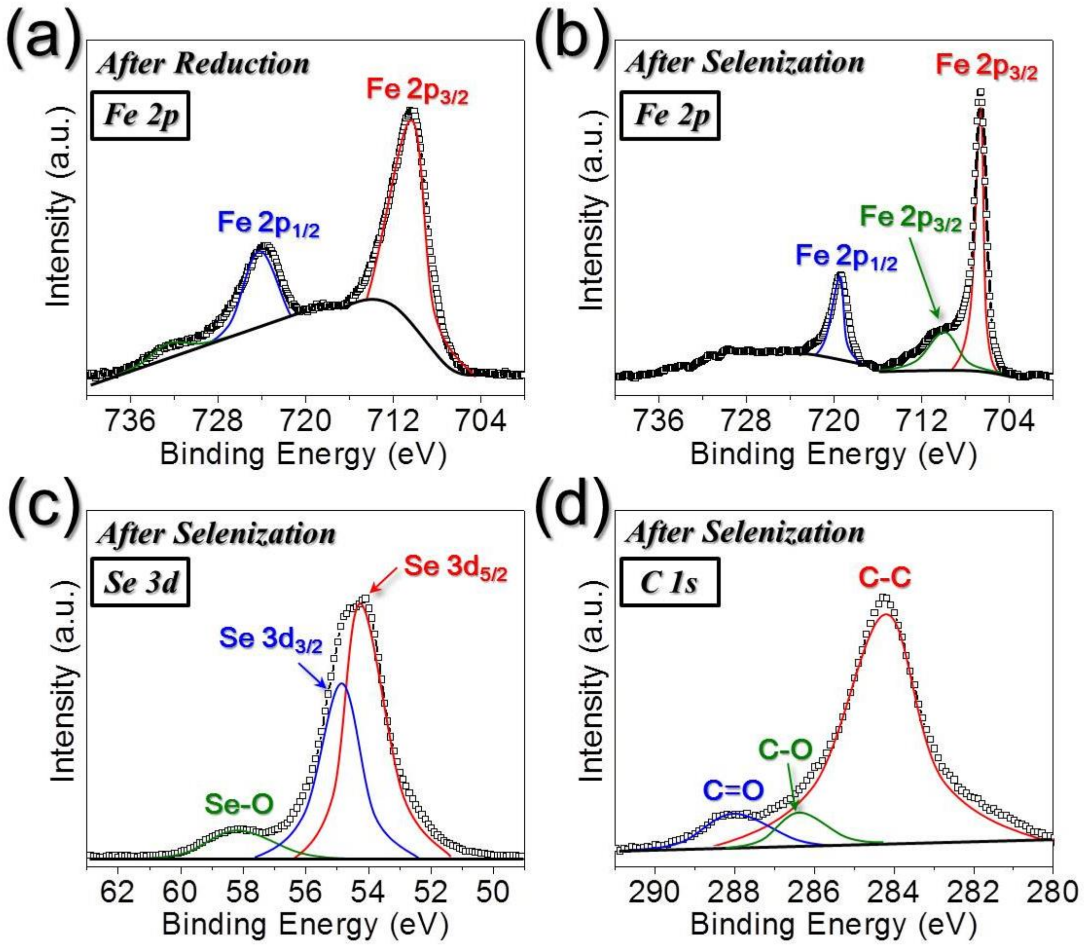

Figure S5. XPS spectra of the (a) Fe-rGO-GC hybrid nanofibers and (b-d) hollow nanosphere $FeSe_2$@GC–rGO hybrid nanofibers: (a,b) Fe 2p, (c) Se 3d, and (d) C 1s.

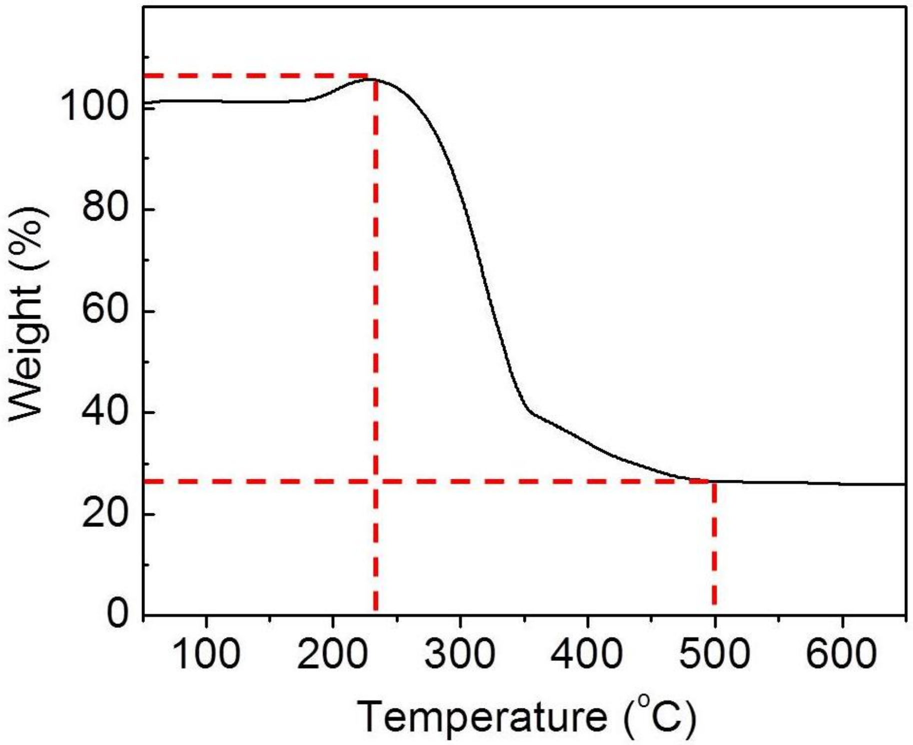

Figure S6. TG curve of the hollow nanosphere $FeSe_2$@GC–rGO hybrid nanofibers obtained after selenization process.

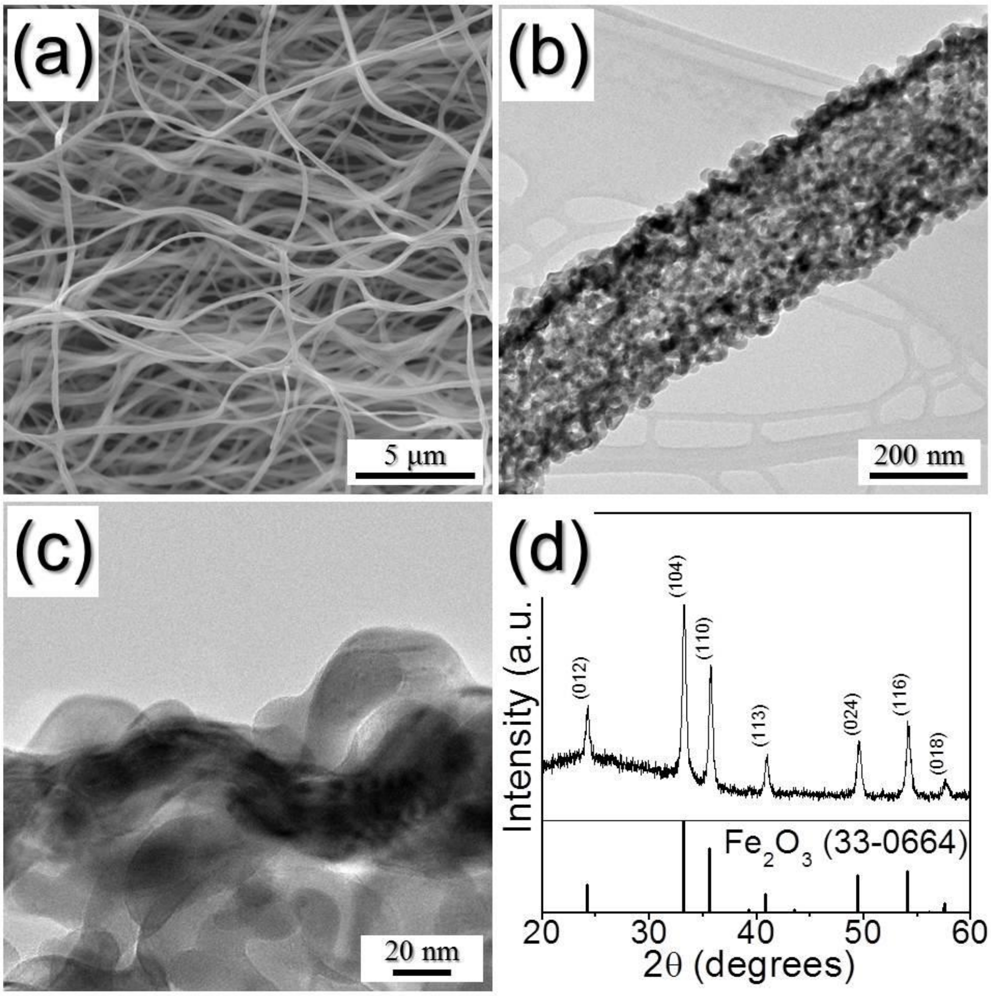

Figure S7. Morphologies and XRD pattern of the bare Fe_2O_3 nanofibers obtained after oxidation process of the $Fe(acac)_3$–PAN-PS composite nanofibers under air atmosphere at 500 ^{o}C: (a) SEM image, (b,c) TEM images, and (d) XRD pattern.

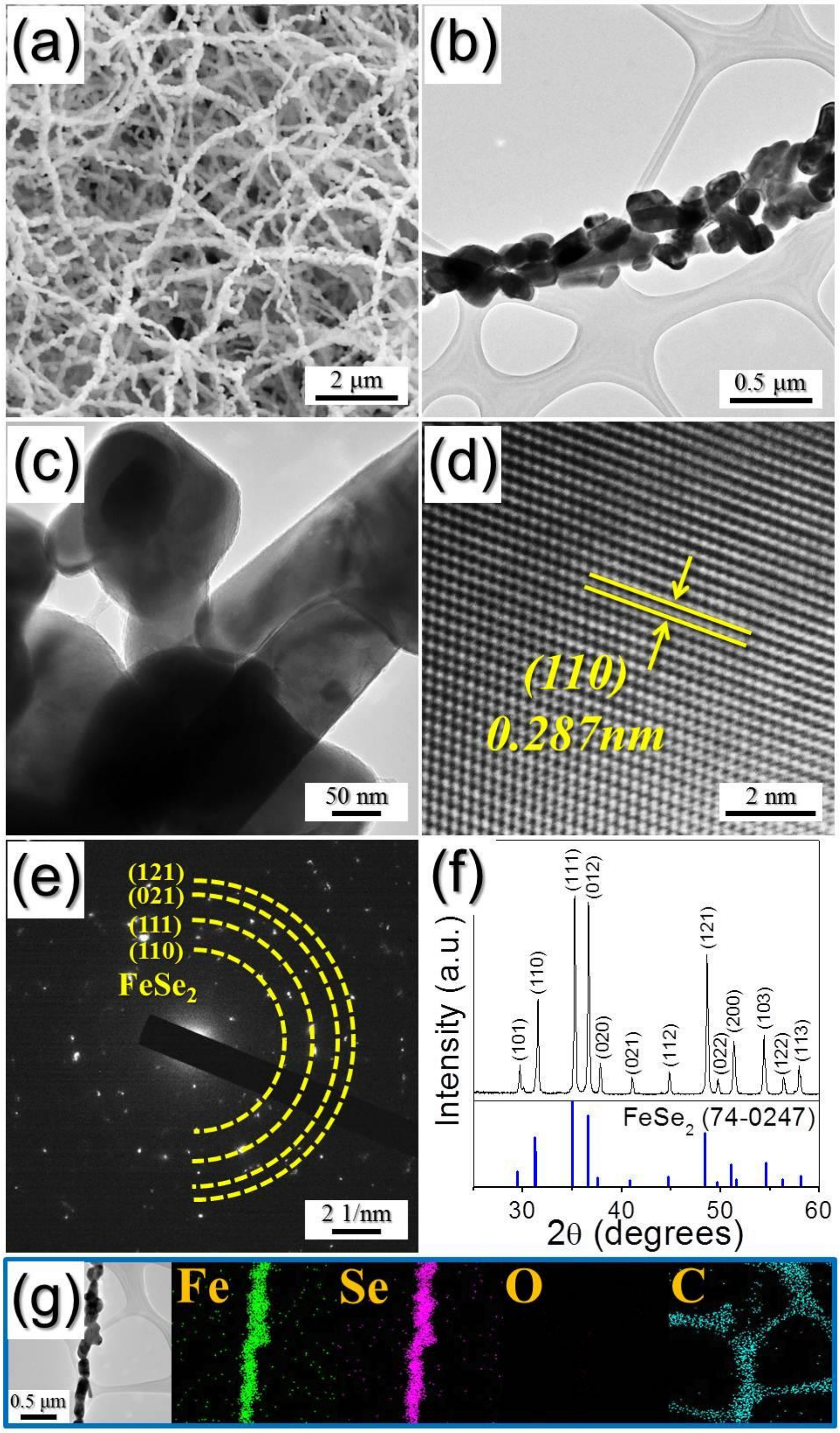

Figure S8. Morphologies, SAED pattern, XRD pattern, and elemental mapping images of the bare $FeSe_2$ nanofibers: (a) SEM, (b,c) TEM images, (d) HR-TEM image, (e) SAED pattern, (f) XRD pattern, and (g) elemental mapping images.

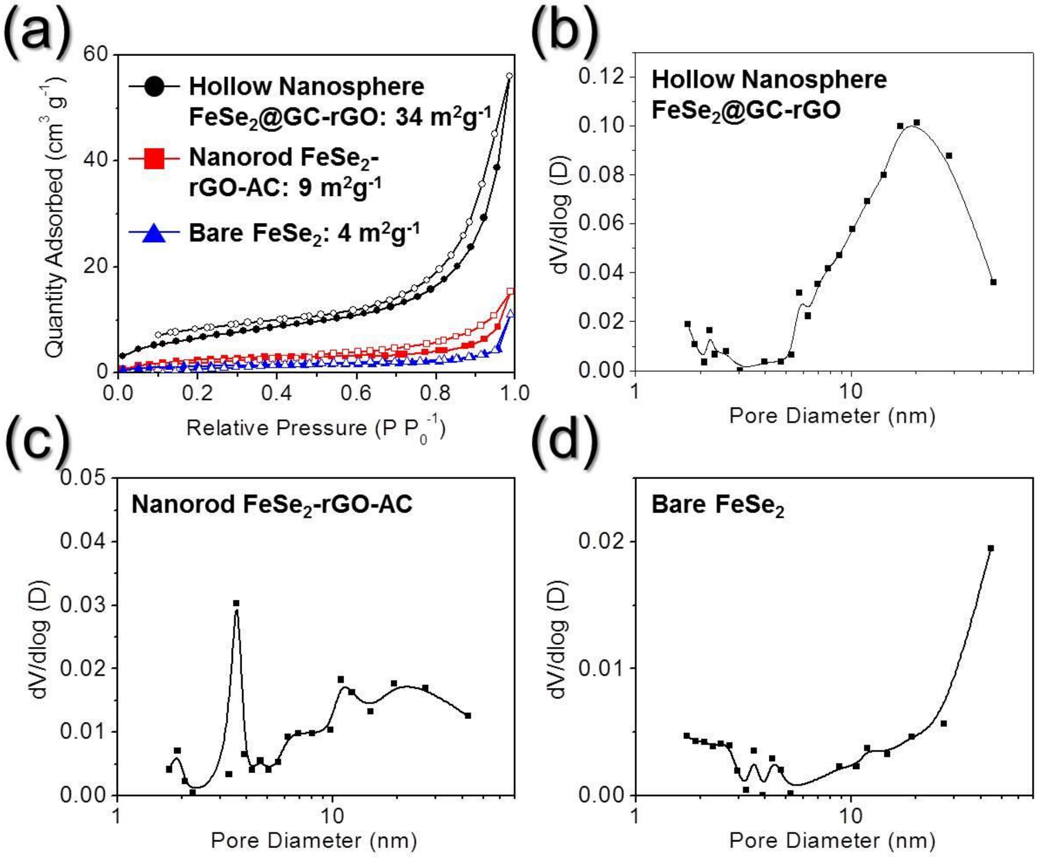

Figure S9. (a) N_2 gas adsorption and desorption isotherms and pore size distributions of (b) hollow nanosphere $FeSe_2$@GC–rGO hybrid nanofibers, (c) nanorod $FeSe_2$-rGO-AC hybrid nanofibers, and (d) bare $FeSe_2$ nanofibers.

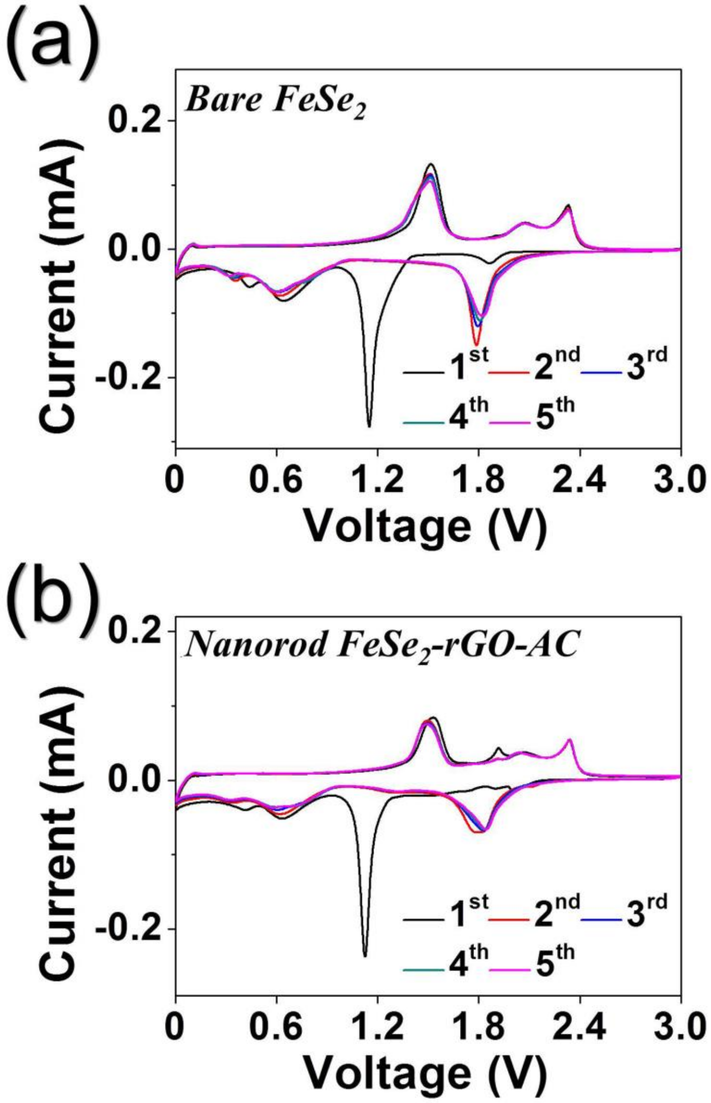

Figure S10. CV curves of the (a) nanorod $FeSe_2$-rGO-AC hybrid nanofibers and (b) bare $FeSe_2$ nanofibers.

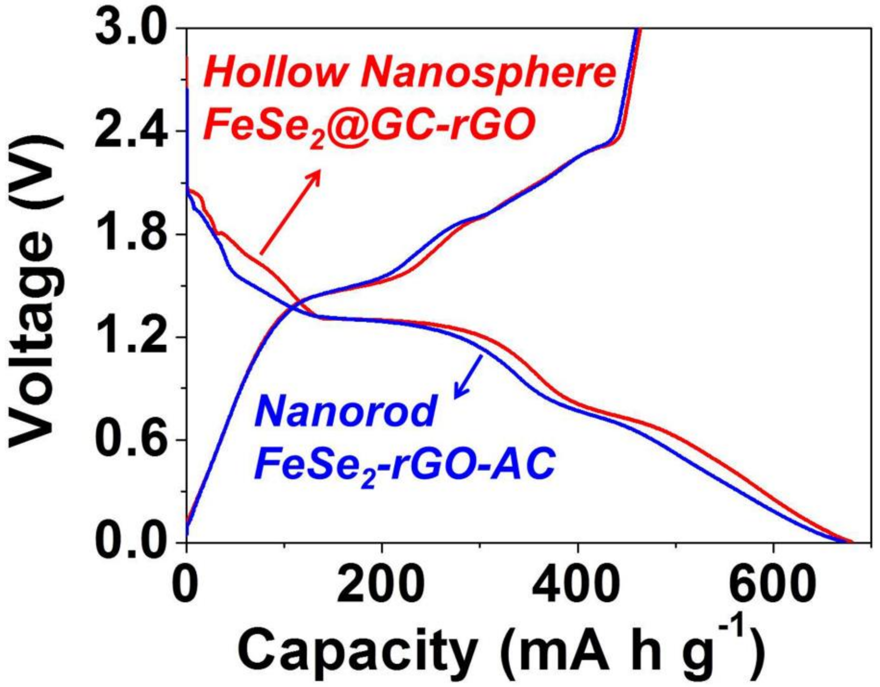

Figure S11. First charge-discharge curves of the hollow nanosphere $FeSe_2$@GC–rGO hybrid nanofibers and nanorod $FeSe_2$-rGO-AC hybrid nanofibers at a current density of 50 mA g^{-1}.

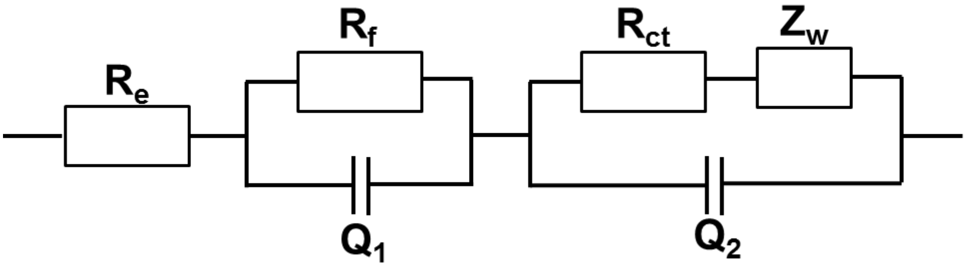

R_e : the electrolyte resistance, corresponding to the intercept of high frequency semicircle at Z_{re} axis

R_f : the SEI layer resistance corresponding to the high-frequency semicircle

Q_1 : the dielectric relaxation capacitance corresponding to the high-frequency semicircle

R_{ct}: the denote the charger transfer resistance related to the middle-frequency semicircle

Q_2 : the associated double-layer capacitance related to the middle-frequency semicircle

Z_w : the Na-ion diffusion resistance

Figure S12. Randle-type equivalent circuit model used for AC impedance fitting.
